# Hydrophilic Surface-Modified PAN Nanofibrous Membranes for Efficient Oil–Water Emulsion Separation

**DOI:** 10.3390/polym13020197

**Published:** 2021-01-07

**Authors:** Evren Boyraz, Fatma Yalcinkaya

**Affiliations:** 1 Faculty of Mechatronics, Technical University of Liberec, Studentska 1402/2, 46117 Liberec, Czech Republic; evren.boyraz@tul.cz; 2 Centre for Nanomaterials, Advanced Technology and Innovation, Technical University of Liberec, Studentska 1402/2, 46117 Liberec, Czech Republic

**Keywords:** nanofibre, nanoparticle, membrane filtration, separation, oily wastewater, PAN, TiO_2_

## Abstract

In order to protect the environment, it is important that oily industrial wastewater is degreased before discharging. Membrane filtration is generally preferred for separation of oily wastewater as it does not require any specialised chemical knowledge, and also for its ease of processing, energy efficiency and low maintenance costs. In the present work, hybrid polyacrylonitrile (PAN) nanofibrous membranes were developed for oily wastewater filtration. Membrane surface modification changed nitrile groups on the surface into carboxylic groups, which improve membrane wettability. Subsequently, TiO_2_ nanoparticles were grafted onto the modified membranes to increase flux and permeability. Following alkaline treatment (NaOH, KOH) of the hydrolysed PAN nanofibres, membrane water permeability increased two- to eight-fold, while TiO_2_ grafted membrane permeability increase two- to thirteen-fold, compared to unmodified membranes. TiO_2_ grafted membranes also displayed amphiphilic properties and a decrease in water contact angle from 78.86° to 0°. Our results indicate that modified PAN nanofibrous membranes represent a promising alternative for oily wastewater filtration.

## 1. Introduction

Contamination of food stuffs is now a major risk to human health, the main causes being contamination due to increasingly widespread usage of chemicals and polymers, accidental or intentional environmental pollution (including water sources), an increase in waste and inadequate treatment of wastewaters. As a result, increasingly strict environmental policies and legislation have been implemented in the EU in order to improve environmental quality, protect Europe’s natural resources and protect the health of citizens. Fulfilling these regulations, however, is challenging, particularly as regards the treatment and clean-up of pollutants. Wastewater treatment, in particular, requires special attention. While almost all living organisms require water to survive, unclean water can cause health problems and even death. Nevertheless, the amount of wastewater produced, and especially oily industrial wastewater, has increased drastically as a consequence of population growth and industrialisation.

Nowadays, membrane technology is generally preferred over other oil/water treatment methods owing to its ease of operation, cost effectiveness, high permeability and lack of chemical additives [1]. Membrane separation is also more efficient than conventional separation techniques due to the specific properties of the membrane itself, which can be improved further through the use of nanofibres, which improve the membrane’s functionality, selectivity and permeability. During separation, the membrane acts as a semi-permeable layer between two faces, with the membrane regulating the flow of liquid from one side to the other while catching colloids, solids or other substances on the membrane’s surface, including the various hydrocarbons that make up oil. Polyacrylonitrile (PAN) is a polymeric material widely used in membrane technology due to its high resistance to chemicals and good membrane performance in aqueous filtration applications [2]. While its hydrophilic properties tend to decrease with fouling, treatment with an alkaline, such as sodium hydroxide (NaOH), improves the chemical stability of PAN, both to common solvents and polar aprotic solvents [3]. Moreover, alkaline treatment causes the membrane to swell somewhat, thereby reducing the pore diameter [4]. Electrospun nanofibre webs are relatively easy to produce using PAN polymers [5,6,7,8] and the resultant nanofibre membranes have an extremely high specific surface area, a highly porous structure and tight pore size [9,10].

In a previous study [11], we modified PAN nanofibre membranes using plasma treatment followed by NaOH treatment to improve membrane hydrophilicity. Under optimal conditions, the permeability of these membranes increased four-fold. In this study, we prepare PAN nanofibres on a semi-industrial scale wire electrospinning system and test their ability to separate an oil-water emulsion. Unlike previous studies [11,12], we modified the PAN nanofibres using alkaline treatment followed by direct grafting of titanium dioxide (TiO_2_) nanoparticles, with no plasma treatment or direct addition of particles to the polymer solution. Two different types of alkaline solution were used to modify the PAN nanofibre membrane and their effects on membrane performance are compared.

While Wang et al. [13] have previously used a TiO_2_ coating on amino-silane modified PAN nanofibres for separation of oily wastewater, their method resulted in high energy and chemical consumption. The surface modification method we propose reduces overall chemical consumption, making the PAN nanofibre layers easier and less complicated to prepare and more suited to bulk production. Further, most previous studies [13,14,15] have prepared membranes at a laboratory-scale only, which can be difficult, requires optimisation and may not be transferrable to real applications. Our own study is novel in that the nanofibre layers were produced using industrial production equipment, with the industrial-scale lamination process in particular resulting in strong membranes predisposed to real-world applications.

## 2. Materials and Methods

### 2.1. Membrane Preparation

**Figure 1:**
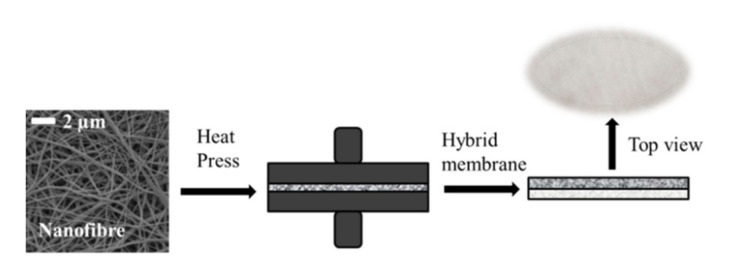
A schematic diagram illustrating membrane preparation.

PAN nanofibres (1 g/m^2^ and 3 g/m^2^) were obtained from the Nanocentre at the Laboratory of nanomaterial application, Technical University of Liberec (Liberec, Czech Republic), while polyethylene terephthalate nonwoven, used as the support layer, was procured from Mogul Nonwovens (Gaziantep, Turkey). The nanofibre and nonwoven layer was then bonded together using a co-polyester adhesive web under heat and pressure (Figure 1). Nanofibre density was determined by the speed of the collector, with a lower speed producing more nanofibres on the membrane surface. Two control membranes were produced for testing, one at 1 g/m^2^ (S1) and the second at 3 g/m^2^ (S2). The higher nanofibre web density was selected for further surface modification (S3–S6) owing to its greater mechanical strength and better handling properties (see Section 3.1; Table 1).

### 2.2. Surface Modification

**Figure 2:**
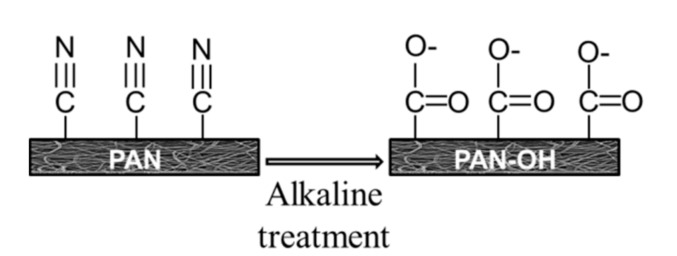
A schematic illustration of the alkaline treatment of PAN membranes.

Surface modification of the membranes was undertaken in two steps. In the first step, the PAN membranes were modified by immersion in NaOH (Penta, s.r.o., Prague, Czech Republic) and potassium hydroxide (KOH; Penta, s.r.o., Prague, Czech Republic) alkaline solutions, the main purpose of this hydrolysis step being to convert surface nitrile groups into carboxylic groups. This takes place in two stages, the first stage being hydrolysis of nitrile groups to generate an amide moiety. The second stage is the removal of –CONH_2_ groups and the formation of carboxylic acids through the addition of a hydroxyl group on the amide [16,17]. In brief, alkaline solution hydrolysis is based on the conversion of –CN groups on the PAN membrane surface into –CONH_2_ and then into –COO– groups (Figure 2).

The alkaline solutions were prepared by dissolving 36 g NaOH in 30 mL of distilled water and 2 g KOH in 20 mL of isopropyl alcohol (Penta s.r.o., Prague, Czech Republic). Both alkaline solutions were mixed for three hours at room temperature, following which, membranes were submerged in the NaOH solution for 48 h and the KOH solution for 1 h (previous experience has shown that the KOH treatment works faster than NaOH [18]). The membranes were then washed with distilled water.

**Figure 3:**
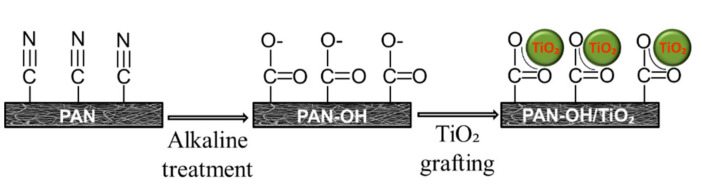
TiO_2_ treatment of PAN membranes.

In the second step, the alkaline treated PAN membranes were dipped into a titanium dioxide (TiO_2_) solution prepared using 40 mL of distilled water and 0.5 g of 20 nm TiO_2_ nanoparticles (Sigma-Aldrich s.r.o., Prague, Czech Republic) to enhance the membrane’s permeability, self-cleaning properties and anti-bacterial properties. The membranes were soaked in the TiO_2_ solution for 24 h, then washed with distilled water using an ultrasonic cleaner for one minute to remove excessive TiO_2_ particles from the membrane’s surface. The reaction between the alkaline treated membrane following TiO_2_ treatment is illustrated in Figure 3. In total, six membranes were prepared for testing (Table 1), 2× alkaline treated (S3 and S5), 2× alkaline and TiO_2_ treated (S4 and S6) and two controls with no treatment (S1 and S2).

### 2.3. Characterisation

A Vega 3SB scanning electron microscope (SEM; TESCAN VEGA, Brno, Czech Republic) was used to characterize membrane surface morphology. The Image-J program (free online program) was used to analyse fibre diameter, with at least 50 measurements undertaken on each fibre. Membrane air permeability was assessed using an ATLAS 128 Air Permeability Tester (SDL ATLAS, Rock Hill, SC, USA) set at 200 Pa and 20 cm^2^. Membrane pore size was measured using a custom-made porometer (Technical University of Liberec laboratory, Liberec, Czech Republic), with minimum, maximum and average pore size obtained based on bubble point measurement [19,20]. The Krüss Drop Shape Analyser DS4 (Krüss GmbH, Hamburg, Germany) was used to characterise membrane water contact angle against distilled water (surface tension 72.0 mN m^−1^). A custom-made device was used to check membrane lamination quality. The membranes were placed between two rings and then pressurised water was applied from one side until the nanofibre layer became delaminated from the supporting layer, the strength of delamination was then recorded based on maximum bursting pressure. Finally, a Nicolet iZ10 fourier transform infrared spectroscope (FTIR; Thermo Scientific, Prague, Czech Republic) was used to characterize changes in the chemical structure of the membrane surface following surface modification.

### 2.4. Emulsion Preparation

**Figure 4:**
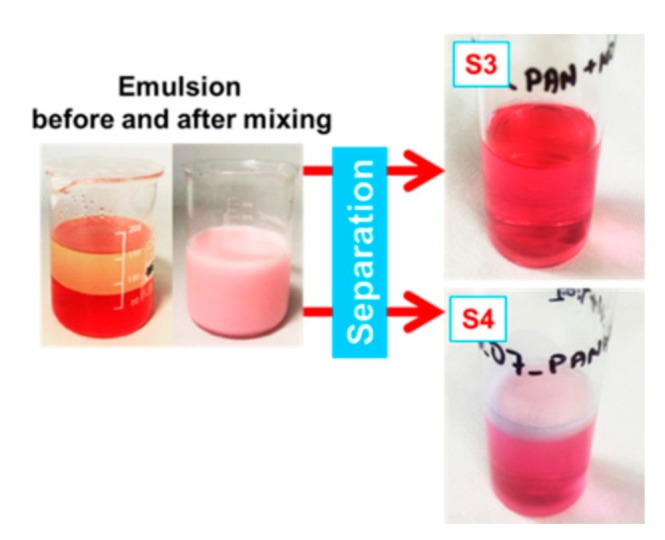
Emulsion before and after mixing and two examples of permeate following separation, illustrating full (top) and partial (bottom) separation.

To produce the experimental emulsion, 100 mL of distilled water and 100 mL of Vita D’or sunflower oil from Lidl Stiftung & Co. KG (Liberec, Czech Republic) were mixed at a ratio of 1:1 *v*/*v* and mixed with a magnetic stirrer (Heidolph Instrument GMBH & Co., Schwabach, Germany). We then added 2 g of non-ionic Triton X-100 (Sigma-Aldrich. s.r.o., Prague, Czech Republic) to reduce oil droplet size and oil droplet surface tension [21], along with a few drops of red food colouring to help in determining the permeate following separation (see Figure 4). The emulsion was then mixed with a magnetic stirrer at 500 rpm for a further 24 h.

**Figure 5:**
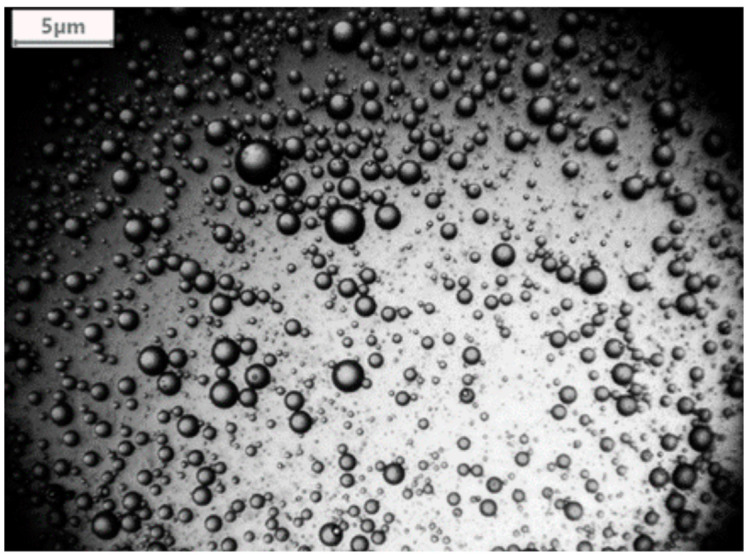
A digital image illustrating the oil droplets within the experimental emulsion.

A Levenhuk C800 NG microscope digital camera, Levenhuk (Prague, Czech Republic) was used to characterize the emulsion (Figure 5). This revealed an average oil droplet diameter of 1058.02 nm ± 345.39 nm. To check whether oil droplet diameter changed over time, the emulsion was left for a few weeks without stirring and then checked under the microscope once again. This revealed no change in oil droplet diameter.

### 2.5. Filtration Test

**Figure 6:**
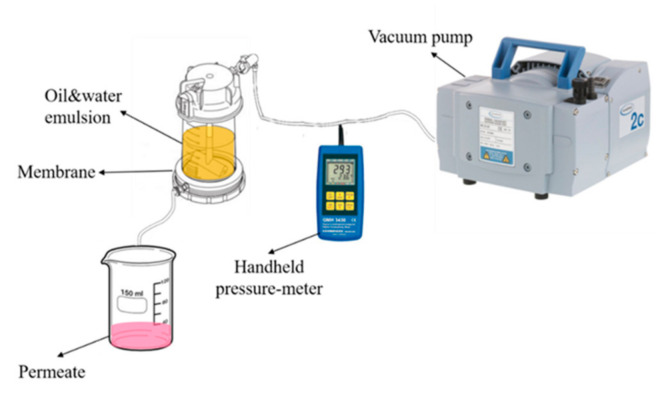
Illustration of the separation system used in this study.

The Amicon 50 mL stirred cell dead-end filtration unit (Millipore Corporation, Billerica, MA, USA) was used to separate the oil/water emulsion, using a 44.5 mm diameter active membrane and a Vacuubrand vacuum pump (VACUUBRAND GMBH + CO KG, Wertheim Germany) set at 0.02 bar pressure. A handheld pressure-meter (GMH-GREISINGER s.r.o., Prague, Czech Republic) was used to control the pressure applied to the separation system (see Figure 6).

Flux (*F*; L·m^−2^·h^−1^) and permeability (*k*; L·m^−2^·h^−1^·bar^−1^) were calculated using the following Equations (1) and (2).

(1) $$F=\frac{1}{A}\frac{dV}{dt}$$

(2) $$k=\frac{F}{p}$$

where *V* is the total volume of the permeate (L), *A* is the active membrane area (m^2^), *t* is the filtration time (hours) and *p* is the transmembrane pressure (bar).

Selectivity was initially assessed based on permeate colour (see Figure 4, right side). The permeate was also checked under a microscope to determine presence of oil droplets. In order to assess the self-cleaning or fouling properties of the membranes, the separation test was repeated three times without changing the membrane with 15 mL of distilled water filtered first, followed by 15 mL of oil/water emulsion, in each separation cycle.

## 3. Results

### 3.1. Membrane Characterisation

**Figure 7:**
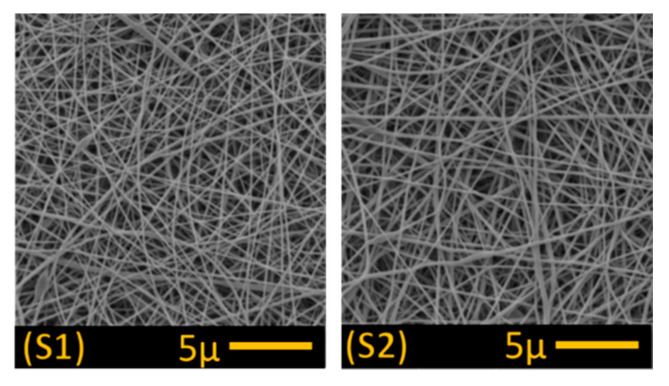
Scanning electron microscope images of the control membranes S1 (1 g/m^2^) and S2 (3 g/m^2^).

**Figure 8:**
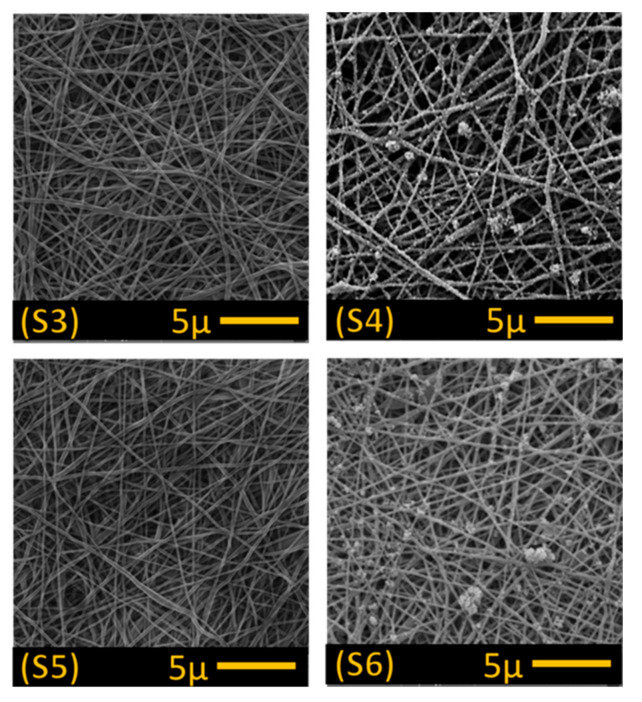
Scanning electron microscope images of the four modified membranes (S3–S6; see Table 1).

SEM images obtained for the unmodified control membranes (S1 and S2; Figure 7) and modified membranes (S3–S6; Figure 8) showed no significant difference in fibre morphology under different nanofibre web densities (see Table 1), suggesting that collector speed has no significant effect on fibre diameter. After modification in alkaline solution, however, fibre diameter increased slightly, and increased still further following TiO_2_ treatment (Table 1). In our modification system, the nitrile groups (–C≡N) were hydrolysed and converted into carboxyl groups (–COO–) following immersion in the alkaline solutions, which improved the surface’s hydrophilic and charging properties. In addition, hydrolysis of PAN resulted in membrane swelling, reducing the pore size and making the membrane surface smoother [4,22]. Functional carboxyl groups attract TiO_2_, causing it to graft onto the membrane surface (Figure 8). This configuration of carboxylic groups with TiO_2_ is known as bidentate chelation, bidentate bridging, and C=O or C–OH monodentate complexation [23,24].

SEM images indicated that TiO_2_ particles were not well distributed on the treated membranes, with clear aggregation on the fibre’s surface (Figure 8; S4 and S6), suggesting that PAN hydrolysis following alkaline treatment was irregular. In a previous study [18], a more regular distribution of TiO_2_ nanoparticles was observed on the surface of polyvinylidene fluoride (PVDF) nanofibres following alkaline treatment as dehydrofluorination of the PVDF surface was more regular, allowing improved attachment of TiO_2_ nanoparticles to hydrophilic –OH groups on the fibres.

**Figure 9:**
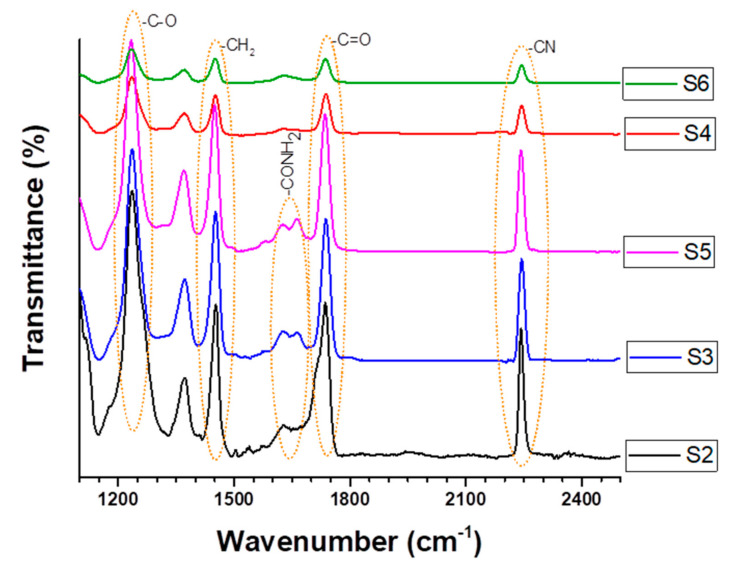
FTIR spectra of PAN membranes with and without modification with TiO_2_ nanoparticles and different alkaline species.

The peaks at 2242 and 1452 cm^–1^ in the FT-IR polyacrylonitrile membrane spectra before and after modification were due to bending of the –CN group and CH_2_, respectively (Figure 9). The peaks decreased slightly in both the modified S3 and S5 membranes, confirming partial (incomplete) hydrolysis and conversion of CN to COOH groups. On the other hand, these same peaks were reduced sharply in all cases following alkaline and TiO_2_ treatment. Following reaction with the alkaline solution, hydrolysis resulted in peaks between 1600 and 1700 cm^−1^, which probably indicates the presence of –COONa and -CONH_2_ groups in the S3 and S5 modified membranes. It has previously been shown that the –CN group on PAN nanofibres can be transformed into amide (–CONH_2_) and carboxylic sodium (–COONa) groups following being hydrolysis in alkaline [25].

The peak at 1735 cm^−1^ indicates C=O stretching. This same peak was also observed in pristine PAN membranes and is attributable to vinyl acetate monomers. The peak increased slightly following alkaline treatment due to the formation of carboxyl groups.

All peaks characteristic of PAN (2242, 1735, and 1452 cm^−1^) in the S4 and S6 samples were significantly reduced following treatment due to the attachment of TiO_2_ nanoparticles to the alkaline-treated membrane surface.

Membrane selectivity, permeability, fouling and rejection are primarily dependant on membrane pore size and operating conditions (pH, temperature, etc.). In this study, all operating conditions were kept the same and all modified samples were preserved in distilled water; thus, any differences are likely to be due to pore size alone. Our results indicated that average membrane pore size decreased as nanofibre web density increased (Table 1), with an initial pore size for control membrane S1 (1 g/m^2^) of 1.74 ± 0.1 µm decreasing to 0.9 ± 0.08 µm in control membrane S2 (3 g/m^2^) due to its higher fibre density and more compact structure. The final pore size following modification (S3–S6) was reduced even further (by around 50%; Table 2), due to fibre swelling following alkaline treatment and TiO_2_ coating the fibres.

As most separation units work under high pressures, a standard membrane must be able to resist damage or bursting under external pressure; hence, we tested bursting pressure to determine the degree of delamination for each layer and to assess lamination process quality. Both S1 and S2 control membranes showed high bursting strength (Table 3), with at least two bars (applied from the rear side) needed to separate the nanofibre layer from the support. In such cases, modification reduced adhesion between the layers, thereby reducing its tensile properties. Based on these results, we consider 90 kPa to be the maximum recommended pressure for backwash cleaning of membranes prepared in this way.

As observed in previous studies [18,26], a higher web density generally results in increased fibre compaction, which reduces air permeability. As such, we undertook an air permeability test following the lamination process in order to assess to what degree the melted adhesive web clogged the nanofibre pores. As expected, the increased density of sample S2 reduced its permeability (Table 3); however, S2 displayed a better bursting strength than S1, giving it greater mechanical strength and better handling properties, hence S2 was chosen for further surface modification. The air permeability of all samples was reduced following modification, presumably due to the reduced pore size caused by fibre swelling after alkaline treatment and adhesion of nanoparticles on the fibre.

To assess the wettability of the membranes, the water contact angle of both modified and unmodified membranes was measured before and after separation took place. Our results showed that the unmodified membranes displayed a lower contact angle after separation (Table 4), probably due to surface contamination and the effects of the non-ionic Triton X-100 surfactant on the membrane. All the modified membranes (S3–S6) displayed surface hydrophilicity (Table 4) due to the formation of the strongly hydrophilic carboxyl group (COOH) on the surface of the membranes. While alkaline treatment alone (S3 and S5) was enough to promote super hydrophilic functionality, previous studies have shown that TiO_2_ also exhibits super hydrophilicity when illuminated under UV light (e.g., [27]); hence, the use of an external source of UV light might be expected to further enhance the super hydrophilic properties of membranes S4 and S6 (TiO_2_ treated). However, in a previous study [28] in which TiO_2_ nanoparticles were grafted onto a membrane surface following plasma and alkaline treatment, there was no apparent impact on membrane separation performance following TiO_2_ treatment with and without UV-light, possibly due to the particle size used, which can affect its photocatalytic activity. In this work, the membranes were prepared in the laboratory and filtration was done under natural sunlight; hence, we were unable to assess the impact of external UV light treatment on the TiO_2_ treated membranes. It should also be noted that membrane contact angle is a function not only of the nanofibre itself but also the components used to construct it. In our samples, it is possible that the adhesive web used in the lamination process may have affected the membrane contact angle, and hence hydrophilicity, by covering the surface of the nanofibres.

### 3.2. Separation Test

One disadvantage of units such as that used in our test is that the membrane in the dead-end filtration unit gradually becomes fouled as contaminants collect on the membrane surface, thereby reducing its permeability. To assess the self-cleaning and selectivity capabilities of each membrane, we repeated the separation process on each membrane three times without changing the membrane between cycles, with each cycle consisting of a wash through with distilled water followed by a feed wash with the oil/water emulsion.

**Figure 10:**
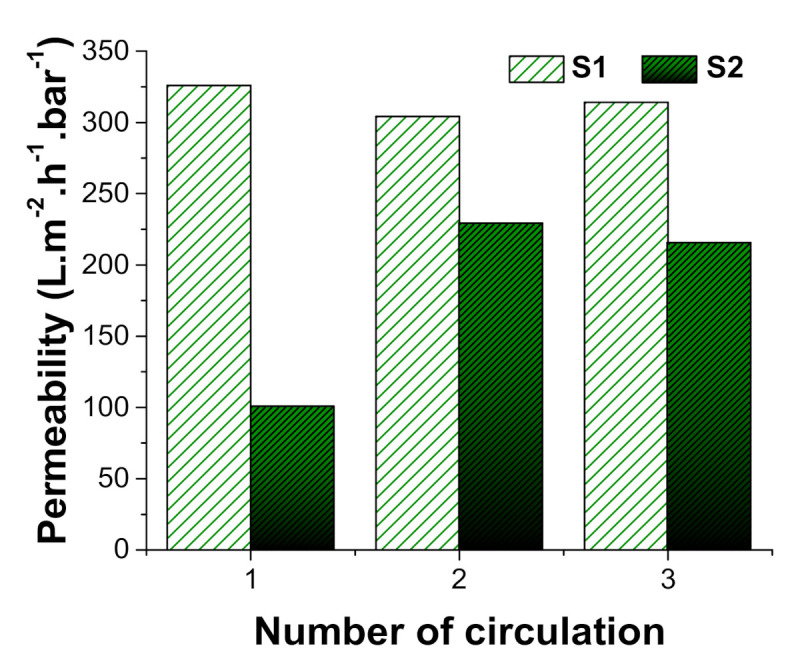
Permeability (oil/water separation) for the two control membranes used in this study (S1 = 1 g/m^2^, S2 = 3 g/m^2^).

As might be expected, a comparison of the two control membranes indicated that membrane S2 (3 g/m^2^) was less permeable that S1 (1 g/m2) owing to its more compact nanofibre web and reduced pore size, with initial readings ca. one third of those for S1 (Figure 10). Similar results were obtained in a previous study using membranes prepared with polyamide 6 (PA6) nanofibres [29], whereby a 1.11 g/m^2^ PA6 membrane displayed permeability almost twice that of a 2.31 g/m^2^ PA6 membrane. While S1 permeability remained virtually unchanged over successive cycles, with just a slight reduction probably caused by fouling, S2 showed an increase in permeability of ca. two-fold over the second and third cycles, most likely due to the surfactant. Nevertheless, S2 permeability remained at around two thirds that of S1. Aside from the first run for S2, permeability readings remained roughly similar over successive cycles (Figure 10), most likely due to the non-ionic Triton X-100 surfactant added at the suspension preparation stage (see also results for ‘wettability’ above). In a previous study, Fane et al. [30] found that the addition of a non-ionic surfactant increased membrane flux by 20% and also made membrane cleaning easier. On the other hand, other studies have reported that surfactant addition can decrease membrane flux due to concentration polarisation and trapping of adsorbate surfactant molecules in the membrane pores [31,32,33,34]. It is possible that these conflicting results for flux and permeability arise from the actual amount of non-ionic surfactant used in the emulsion.

**Figure 11:**
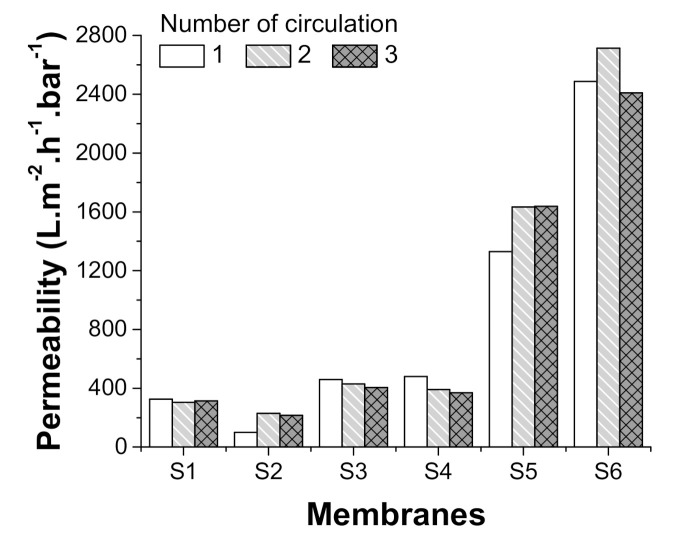
Permeability (oil/water separation) of all membranes used in this study.

While treatment with alkaline improved permeability in both test membranes, there was a clear difference depending on the alkaline used, with permeability of those treated with NaOH (S3) improving just two-fold while those treated with KOH (S5) improving ca. eight-fold (Figure 11). This difference in the degree of hydrolysis, which is due to differences in the attack strength of the hydroxyl group, was also confirmed in the study of Zhang et al. [35], who recorded clear differences in hydrolysis levels between membranes treated with KOH, NaOH and lithium hydroxide (LiOH), with the degree of hydrolysis mainly in line with the order of basicity intensity.

In addition to increasing membrane hydrophilicity, alkaline treatment has previously been shown to improve membrane chemical resistance to common organic solvents, including DMF (*N*,*N*-Dimethylformamide), DMSO (dimethyl sulfoxide) and NMP (*N*-Methyl-2-pyrrolidone) [3]. Moreover, by decreasing the diameter of PAN fibres to the nano-scale, fibre specific surface area is increased, increasing the amount of carboxyl (–COOH) groups available on the surface [25].

In our own test, the KOH treated membrane (S3) not only showed better flux and permeability than the NaOH membrane (S5), its membrane permeability increased over successive cycles, while that of the NaOH membrane decreased (Figure 11). Roughly similar results were recorded for the NaOH membrane following TiO_2_ nanoparticle grafting (S4), with permeability remaining similar (ca. two-fold increase) and decreasing over successive cycles (Figure 11). The TiO_2_-grafted KOH membrane (S6), however, showed a further increase in permeability, with a ca. thirteen-fold increase over the non-treated control membrane (S2) and a ca. one-and-a-half-fold increase over the membrane treated with KOH alone (S5; Figure 11). As such, a positive effect from grafting TiO_2_ nanoparticles to alkaline treated membranes was only observed in the case of KOH treatment, most likely as the greater increase in hydrolysis resulting from KOH treatment allowed more TiO_2_ nanoparticles to attach to the membrane surface.

As shown previously by Yalcinkaya et al. [11], laminated PAN membranes are hydrophilic, i.e., they repel oil when filtering oil/water mixtures; further, as our own results show, the surfaces of alkaline-treated membranes also show enhanced hydrophilic/oleophobic properties when filtering oil/water emulsions (Table 5). Surprisingly, however, further enhancement with TiO_2_ reduced membrane selectivity, with oil droplets being found in the permeate from both KOH and NaOH membranes after separation (Table 5). There are a number of possible reasons for this apparent increase in oleophilicity. Firstly, TiO_2_ is likely to have increased surface roughness. Shuai et al. [36], for example, found that addition of TiO_2_ nanoparticles to the surface of a polyurethane sponge increased surface roughness and hydrophobicity, while Gao et al. [37] noted that increased surface roughness caused by grafting TiO_2_ particles to the surface of cotton increased the material’s oleophilic properties. As stated earlier, UV illumination of TiO_2_ particles has the potential to significantly increase surface hydrophilicity and oleophilicity (e.g., see [27]), making them highly amphiphilic. Wang et al. [38] noted that changes in wettability occurred on both anatase and rutile TiO_2_ surfaces in the form of either polycrystals or single crystals, independent of TiO_2_ particle photocatalytic activity, and that this change in wettability resulted in zero contact angles for both water and oily liquids [39]. The high amphiphilicity of this TiO_2_ surface material was maintained, even after storage in a dark place for a few days. In our experiment, the filtration experiments took place under sunlight and, as such, natural light UV may have increased the amphiphilicity of our modified membranes; however, the low levels from sunlight alone do not appear to have been sufficient to maintain the amphiphilic surface.

Our results indicate that alkaline treatment improves PAN membrane hydrophilicity and flux and deceases overall pore size, while additional grafting of TiO_2_ nanoparticles decreases membrane selectivity but improves permeability and fouling resistance. We conclude that separation between the hydrophilic and oleophilic phases accounts for the amphiphilic character of the TiO_2_ grafted membrane surfaces used here, with the hydrophilic part improving membrane flux and permeability and potentially reducing the cake layer that forms on the membrane surface. It is likely that the hydrophobic side becomes attached to the hydrophobic membrane matrix, and that the hydrophilic side then becomes stabilised on the surface, resulting in reduced interaction between the foulant and membrane surface. As such, surface modification of the membrane helped improve the membrane’s anti-fouling properties while maintaining its high flux and rejection properties, with the result that the membrane showed high permeability and self-cleaning properties, even when faced with high concentrations of high-viscosity oil in emulsion.

Numerous studies have shown that membrane separation performance against oil-water emulsions varies greatly depending on the amount of oil in emulsion, the type of oil (low viscosity, high viscosity), droplet size, amount of surfactant, type of surfactant and pressure applied (See Table 6). Note, however, that many of the studies presented in Table 6 used low-viscosity oils, whereas our emulsion used high-viscosity kitchen oil, which can cause membrane fouling immediately. As such, many of these studies cannot be compared directly with ours but are presented for information purposes only.

## 4. Conclusions

In this study, we successfully prepared PAN nanofibrous hybrid membranes of two densities and tested membrane permeability against an oil/water suspension. Based on the results, we chose one of the membranes for further modification with two different alkaline solutions (NaOH, KOH) and TiO_2_ nanoparticle grafting. Each membrane was then subjected to three cycles of oil/water feed and distilled water flushing, with membrane permeability and membrane fouling assessed after each cycle.

The results indicated that:

Membranes produced with a lower density nanofibre web (S1; 1 g/m^2^) displayed a more open structure and higher water permeability but lower mechanical strength, while those produced with a higher density nanofibre web (S2; 3 g/m^2^) displayed a less open structure and slightly lower water permeability but had higher mechanical strength and were easier to handle. Based on this, the higher density membrane was chosen for further modification.

After alkaline treatment, the PAN nanofibres were hydrolysed with a consequent reduction in pore size, and both displayed hydrophilic/oleophobic characteristics. Permeability improved in both test membranes; however, there was a clear difference depending on the alkaline used, with permeability of the NaOH-treated membrane improving two-fold and that for the KOH-treated membrane improving ca. eight-fold

While grafting of TiO_2_ nanoparticle to the membranes had little effect on the NaOH-treated membrane, the KOH membrane showed a clear increase in permeability, with a ca. thirteen-fold increase over the non-treated control membrane and a ca. one-and-a-half-fold increase over the membrane treated with KOH alone. As such, TiO_2_ treatment improved flux and permeability, increased the membrane’s amphiphilic properties and improved the fouling resistance characteristics of the KOH-treated membrane, with the membrane also showing both hydrophilic and oleophilic phases due to the amphiphilic character of TiO_2_. Such amphiphilic surfaces have many practical applications, including use as surface cleaners or biomembranes.

Our results suggest that there is a correlation between contact angle, hydrophilicity, fouling with oily waste and permeability, with more hydrophilic materials resulting in a reduced contact angle, less fouling and higher permeability.

Based on these results, it would appear that a one-step KOH alkaline modification is sufficient to improve membrane flux and permeability without reducing selectivity. On the other hand, a two-step modification using KOH and TiO_2_ nanoparticles helps improve the membrane’s amphiphilic surface characteristics.

## Figures and Tables

**Table 1:** Summary of the different PAN nanofibre membrane preparations used in this study.

Polymer	Abbreviation	Density (g/m^2^)	Modification	Fibre Diameter (nm)
PAN	S1	1	-	143.76 ± 30.83
	S2	3	-	168.43 ± 26.52
	S3	3	NaOH	165.38 ± 36.36
	S4	3	NaOH + TiO_2_	207.03 ± 56.44
	S5	3	KOH	170.43 ± 42.67
	S6	3	KOH + TiO_2_	179.12 ± 34.56

**Table 2:** Mean pore size unmodified and modified PAN nanofibrous membranes.

Abbreviation	Mean Pore Size (µm)
S1	1.74
S2	0.90
S3	0.45
S4	0.45
S5	0.44
S6	0.38

**Table 3:** Bursting strength and air permeability of the two control membranes (S1 = 1 g/m^2^, S2 = 3 g/m^2^).

Sample Code	Bursting Strength [kPa]	Air Permeability [l/m^2^/s]
S1	189.33 ± 34.32	6.06 ± 0.85
S2	241 ± 20.05	4.07 ± 0.57
S3	149 ±17.00	3.45 ± 0.42
S4	148 ± 14.50	3.64 ± 0.09
S5	125 ± 46.5	2.51 ± 0.01
S6	90 ± 18.00	2.62 ± 0.96

**Table 4:** Contact angle (CA) of control and modified membranes before and after separation.

Sample	CA Before Separation (°)	CA After Separation (°)	Image (Before Separation)
S1	73.93 ± 2.61	39.90 ± 3.47	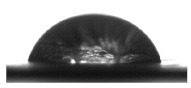
S2	78.86 ± 3.92	69.80 ± 1.50	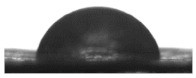
S3	0	0	-
S4	19 ± 6.65	0	-
S5	0	0	-
S6	0	43.85 ± 4.30	-

**Table 5:** Selectivity of the PAN nanofibrous membranes used in this study.

Sample	Permeate	O/W Ratio in Permeate (*v*/*v*) %
S1	water	0/100
S2	water	0/100
S3	water	0/100
S4	oil + water	10/90
S5	water	0/100
S6	oil + water	10/90

**Table 6:** A comparison of oil-water emulsion separation performance for the modified membranes produced in this study and those reported in the literature.

Membrane	Feed	Pressure Applied (Bar)	Flux-Permeability	References
PAN–OH, TiO_2_ grafted PAN-OH	50/50 *v*/*v* oil/water emulsion	0.02	600–2600 L/(m^2^ hbar) for emulsion	This work
TiO_2_ coated amino-silane modified PAN nanofibre	1/1000 *wt*/*wt* oil/water emulsion	0.01	Up to 2000 L/(m^2^ h) for emulsion	Wang et al. [13]
Polyacrylonitrile/polyaniline composite	1000 ppm emulsion	0.5–2.3	~2000 L/(m^2^ h) for emulsion at 0.5 bar	Shakiba et al. [15]
HPAN-PEI-PFOS	1 g/L oil/water emulsion	0.5–1	~220 L/(m^2^ h) for emulsion at 0.5 bar	Yu et al. [40]
Perfluoroalkyl coated polyacrylonitrile	1000 ppm emulsion	1	~150 L/(m^2^ h) for emulsion at 1 bar	Zhao et al. [41]
PAN@SiO2 nanofibre membrane	1/100 *v*/*v* oil/water emulsion	0.01	1000–4000 L/(m^2^ h)	Ying et al. [42]
PAN/HPEI/PDA nanofibre membrane	1/100 *v*/*v* oil/water emulsion	0.1–0.2	~1200 L/(m^2^ h)	Wang et al. [43]
Polyvinyl acetate-coated electrospun nylon 6/SiO2	1000 mg/L oil in water	0.28	~2500 L/(m^2^ hbar)	Islam et al. [44]
Commercial PSf	1000 mg/L oil in water	0.28	~800 L/(m^2^ hbar)	Islam et al. [44]
Commercial PVDF	1000 mg/L oil in water	0.28	~200 L/(m^2^ hbar)	Islam et al. [44]

## Data Availability

Not applicable.
